# Cancer risk in patients with alopecia areata: a nationwide population‐based matched cohort study

**DOI:** 10.1002/cam4.1448

**Published:** 2018-03-25

**Authors:** Chih‐Chiang Chen, Yun‐Ting Chang, Han‐Nan Liu, Yi‐Ju Chen

**Affiliations:** ^1^ Institute of Clinical Medicine National Yang‐Ming University Taipei Taiwan; ^2^ Department of Dermatology National Yang‐Ming University Taipei Taiwan; ^3^ Department of Dermatology Taipei Veterans General Hospital Taipei Taiwan; ^4^ Department of Dermatology Taichung Veterans General Hospital Taichung Taiwan

**Keywords:** Alopecia areata, autoimmune disorder, breast cancer, cancer risk, kidney cancer, lymphoma, urinary bladder cancer

## Abstract

Alopecia areata (AA) is an organ‐specific autoimmune disorder. Defective immune system related disorders are prone to increase the risk of cancer formation. However, the association among AA and variety of cancer types had never been studied. A nationwide population‐based matched cohort study was conducted to evaluate the cancer risk in patients with AA. Records from Taiwan National Health Insurance Research Database were analyzed. Cases of AA from 1997 to 2013 and cancers registered in the catastrophic illness profile from the same time period were collected. The standard incidence ratio (SIR) of each cancer was calculated. In total, 2099 cancers among 162,499 patients with AA and without prior cancers were identified. The overall cancer risks in AA patients were slightly decreased, especially among male subjects (SIR: 0.89). Refer to individual cancer, the cancer risk of nonmelanoma skin cancer (NMSC) (SIR: 0.59), upper GI cancer (SIR: 0.70), liver cancer (SIR: 0.82), uterine, and cervix cancer (SIR: 0.84) were significantly lower in patients with AA. In contrast, AA patients were inclined to have lymphoma, breast cancer, kidney, and urinary bladder cancer with the SIR of 1.55, 2.93, and 2.95, respectively. Age stratified analyses revealed female AA patients younger than 50 years old have even higher risk of breast cancer (SIR: 3.37). Further sensitivity analysis showed similar results after excluding major autoimmune disorders. Cancer risk in AA patients is organ specific, and it is not associated with the underlying autoimmune disorders in patients with AA.

## Introduction

Alopecia areata (AA) is an organ‐specific autoimmune disorder which characterized as sudden onset, specific pattern of hair loss. It typically presents with oval or round, well‐circumscribed, bald patches with a smooth surface in a diffuse distribution. The incidence of AA varied from 0.7% to 3.8% according to the hospital‐based studies from different countries 1, 2. Childhood onset of AA is thought to be a poor prognostic factor. Different onset age of AA leads to distinct clinical consequences but the underlying mechanisms are elusive.

Recent studies demonstrated that infiltration of cytotoxic subset of CD8+NKG2D+ T cells around human AA hair follicles along with a concomitant upregulation in the follicle itself of the “danger signals” ULBP3 3 and MICA 4, two NKG2D ligands (NKG2DLs) due to autoimmunity activation is the major etiology of AA. Giving that oncogenesis is affected by immune system tremendously 5, it raises interest to study how the dysregulated immune system in AA influences cancer development. One hypothesis is that the hyperactivated immune system could suppress cancer cells. Conversely, the chronic inflammation is also considered a predisposing factor for cancers 6. According to these theories, it is highly possible that patient with AA may be associated with the occurrence of malignancy.

Alopecia areata had been found to have comorbidity with many immune related disorders, including systemic lupus erythematosus (SLE), rheumatoid arthritis (RA), thyroid disease, vitiligo, psoriasis, and atopic dermatitis based on its autoimmune condition in essence 7. Refer to the relationship with cancer, to the best of our knowledge, there was only one research discussed the risk of skin cancers in patients with AA 8. However, the case number is small and the association between AA and malignancies other than skin cancers was not addressed. In this study, we conducted a nationwide study to evaluate the cancer risk in patients with AA.

## Methods

### Study design

We conducted a nationwide cohort study by retrieving information from all patients with a diagnosis of AA from Taiwan's National Health Insurance Research Database (NHIRD). The NHIRD has been utilized extensively in epidemiologic studies in Taiwan 9. It consists of detailed healthcare data from more than 25 million enrollees, representing more than 99% of Taiwan's entire population. In this database, the diagnostic codes are in the format of the International Classification of Diseases, Revision 9, Clinical Modification (ICD‐9‐CM) with diagnoses made by board‐certified physicians in the corresponding specialties. Personal information including body weight, height, family history, laboratory examination results, lifestyle, and social habits such as smoking or alcohol use was not available from the NHIRD. The data set used in this study consists of de‐identified secondary data released to the public for research purposes. This study was approved by the ethical review board of Taipei Veterans General Hospital, Taipei, Taiwan.

### Study cohorts

All patients with a primary diagnosis of AA (ICD‐9‐CM code 704.01) for the first time were eligible for inclusion in this study. We included only those subjects who had received a diagnosis of AA by dermatologists. We identified a total of 162,499 patients with a diagnosis of AA between 1997 and 2013, which corresponds to an estimated prevalence of 0.7%, consistent with our previous study 7. Among them, 1179 patients had a diagnosis of malignant diseases (ICD 9 140–208.91) before the index date.

### Identification of cancer cases

The diagnostic codes of malignancies were defined as those from 140 to 208.91 in the ICD‐9 CM format. The outcomes of cases were defined as those who had malignancies after 6 months of the diagnosis of AA. All cancer cases were obtained from the Registry of Catastrophic Illness Database, a subpart of NHIRD. The insured who suffer from major diseases can apply for a catastrophic illness certificate which grants exemption from co‐payment. Cancer is statutorily included in the catastrophic illness category. Both outpatient and inpatient claims of beneficiaries with a catastrophic illness registry were collected in the catastrophic illness profile and were distributed as a package.

To apply for a cancer catastrophic illness certificate, cytological or pathological reports or evidence such as additional laboratory and image studies supporting the diagnoses for cancers, like results from tumor marker surveys, X ray, bone scan, CT scan or MRI scan, should be provided. We excluded those with in situ malignancies because these in situ malignant diseases were not issued for a catastrophic illness certificate.

### Cancer risk analysis

All enrolled study subjects were followed up until a first time diagnosis of cancer (except malignancy in situ or metastasis cancers), death, the end of follow‐up in the medical records, the end of the observation period, or the end of 2013. After excluding those with prior malignant diseases, standardized incidence rate ratios (SIRs) of cancers were analyzed. Stratified analyses according to age at diagnosis and gender were conducted.

### Statistical analysis

The demographic data of the study population were first analyzed. Follow‐up for each patient began at the date of diagnosis of AA and ended at the date of censorship, that is, the date of diagnosis of cancer, death, or the end of follow‐up period and was measured in numbers of years. We examined the association between AA and specific cancer types with standardized incidence ratio (SIR). SIR was calculated as follows: the number of cancer cases that arose among AA patients divided by the expected number of cancer cases according to national age‐specific, gender‐specific, and period‐specific cancer rates. Yearly reports of cancer rates were obtained from Taiwan National Cancer Registry. To assess the age effect on the relative risk for malignancies, we analyzed the relative risk among those aged younger than 50 and greater than 50 years at AA diagnosis.

The SAS statistical package (SAS System for Windows, version 9.4; SAS Institute, Cary, NC) was used to perform the statistical analysis of the data in this study.

## Results

In total, 2099 cancers among 162,499 patients with AA and without prior cancers were identified during 1997 to 2013 in Taiwan. The average time period to the appearance of a new cancer was estimated to be 5.14 ± 3.21 months. AA is seen slightly more often in females. The mean ± SD age at diagnosis of AA was 32.3 ± 14.8 years, and most AA patients are younger than 50 years old (87.5%) (Table 1). Female breast cancer (18.82%) is the most common malignancy appeared in AA patients, followed by colon (12.91%), lung (9.77%), and liver (9.15%).

**Table 1:** Demographic data of the patients with AA enrolled in our study

Alopecia areata	Data
Number of Patient	162,499
Cancer case number (%)	2099 (1.29)
Age (median)	30.90 (22.11–42.25)
Age (mean ± SD)	32.29 ± 14.84
<50 (%)	142,229 (87.53)
≥50 (%)	20,270 (12.47)
Gender	
Male	77,951 (47.97)
Female	84,548 (52.03)
Follow‐up year (Mean ± SD)	6.76 ± 3.64
Follow‐up year (Median)	6.52 (3.55–9.87)

The SIRs of cancers in patients with AA are listed in Table 2. The overall cancer risk in patients with AA was slightly lower than that in the general population though there is no significant difference (SIR: 0.96, 95% CI: 0.91–1.00). When stratified by gender, the cancer risk was decreased in male patients (SIR: 0.89, 95% CI: 0.85–0.93) but no difference in female patients (SIR: 1.02, 95% CI: 0.97–1.06).

**Table 2:** The standardized incidence ratio of total cancer risk in patients with alopecia area, stratified by gender and age

AA patients	Cancer	Expected	SIR	95% CI
Total	2099	2197.29	0.96	0.91–1.00
<50	1183	1288.39	0.92	0.88–0.96
≥50	916	908.90	1.01	0.96–1.05
Male	947	1066.25	0.89	0.85–0.93
<50	513	602.36	0.85	0.82–0.89
≥50	434	463.89	0.94	0.90–0.98
Female	1152	1131.04	1.02	0.97–1.06
<50	670	686.03	0.98	0.93–1.02
≥50	482	445.02	1.08	1.04–1.13

Table 3 demonstrated the SIR of specific cancer in AA patients. The risks of nonmelanoma skin cancer (NMSC; SIR: 0.59), upper GI cancer (SIR: 0.70), liver cancer (SIR: 0.82), uterine, and cervix cancer (SIR: 0.84) were significantly decreased in AA patients than general population (Table 3). In contrast, three cancer types were found to have elevated risk in AA patients including female breast cancer (SIR: 2.93), kidney and urinary bladder cancer (sir: 2.95) as well as lymphoma (SIR: 1.55). Further stratified analyses by gender demonstrated that female patients (SIR: 3.24) had higher risk of kidney and urinary bladder cancer than male patients (SIR: 2.81). Another interesting finding is that only female patient (SIR: 1.55) had increased risk of lymphoma (Table 3).

**Table 3:** The standardized incidence ratio of specific cancer risk in patients with alopecia area, stratified by gender

	All			Female			Male		
	*N*	SIR	95% CI	*N*	SIR	95% CI	*N*	SIR	95% CI
Nonhematologic cancer	1993	1.10	1.05–1.15	1092	1.26	1.19–1.34	901	0.95	0.89–1.01
Oral cavity	79	1.03	0.80–1.25	11	0.93	0.38–1.48	68	1.05	0.80–1.29
Nasopharynx and Pharynx	80	0.96	0.75–1.18	21	1.06	0.61–1.51	59	0.93	0.70–1.17
Upper GI	136	0.70	0.58–0.82	50	0.76	0.55–0.97	86	0.67	0.53–0.82
Colon	271	0.99	0.87–1.10	129	0.97	0.80–1.14	142	1.00	0.84–1.17
Liver	192	0.82	0.71–0.94	56	0.78	0.58–0.98	136	0.84	0.70–0.98
Lung	205	1.13	0.98–1.29	101	1.14	0.92–1.36	104	1.13	0.91–1.34
Thymoma	12	1.29	0.56–2.02	7	1.70	0.44–2.95	5	0.96	0.12–1.81
Connective tissue cancer and bone	21	0.99	0.57–1.41	11	1.12	0.46–1.78	10	0.88	0.33–1.42
Skin, melanoma	8	1.54	0.47–2.61	6	2.21	0.44–3.98	2	0.81	0.00–1.93
Skin, NMSC	30	0.59	0.38–0.80	19	0.76	0.42–1.10	11	0.42	0.17–0.68
Female breast	395	2.93	2.64–3.22	395	2.93	2.64–3.22	0	–	–
Male breast	1	1.06	0.00–3.15	0	–	–	1	1.06	0.00–3.15
Uterine and cervix	150	0.84	0.70–0.97	150	0.84	0.70–0.97	0	–	–
Prostate	59	1.26	0.94–1.58	0	–	–	59	1.26	0.94–1.58
Kidney and urinary bladder	113	2.95	2.41–3.50	41	3.24	2.25–4.24	72	2.81	2.16–3.46
Thyroid	101	1.10	0.89–1.32	76	1.05	0.81–1.28	25	1.31	0.79–1.82
Hematologic cancer	112	1.19	0.97–1.41	64	1.43	1.08–1.78	48	0.96	0.69–1.24
Lymphoma	75	1.55	1.20–1.90	45	1.92	1.36–2.48	30	1.21	0.78–1.64
Leukemia	37	0.80	0.54–1.06	19	0.90	0.49–1.30	18	0.72	0.39–1.05

To evaluate the age effect in the three cancer types with greater SIRs, we further stratified the SIR in breast female cancer, kidney and urinary bladder cancer, lymphoma by age. Our results demonstrated that AA patients younger than 50 years have greater risk to get female breast cancer than older ones (SIR: 3.37 vs. 2.25; Table 4).

**Table 4:** Specific cancer for SIR by age stratification

	Cancer	Expected	SIR	95% CI
Female breast cancer	395	134.73	2.93	2.64–3.22
Age (year)				
<50	276	81.80	3.37	2.98–3.77
≥50	119	52.93	2.25	1.84–2.65
Kidney and urinary bladder cancer	113	38.28	2.95	2.41–3.50
Age (year)				
<50	43	13.80	3.12	2.18–4.05
≥50	70	24.48	2.86	2.19–3.53
Lymphoma	75	48.33	1.55	1.20–1.90
Age (year)				
<50	47	29.74	1.58	1.13–2.03
≥50	28	18.59	1.51	0.95–2.06

To determine whether the underlying autoimmune disorders in AA patients might affect the cancer risk, we conducted further sensitivity analysis by exclusion of the patients with autoimmune disorders (including SLE, RA, sicca syndrome, dermatomyositis, scleroderma, pemphigus, and inflammatory bowel disease (IBD)), and the SIR results were similar before and after adjustment (Table S1).

## Discussion

Alopecia areata is the highest prevalent autoimmune human disease presenting as patterned nonscarring hair loss. The prevalence of AA is variable, ranging from 0.2% in the USA to 1.3% in Greece and 2.5% in Japan 10, 11. Although the pathophysiology of AA is not fully understood, breaking down of the immune privilege of the hair follicles followed by autoimmune attack was thought to be the leading cause of AA recently 3, 12. Regarding as an organ‐specific autoimmune disorder, AA is reported to be associated with variety of autoimmune disorders 7, 13. As disease progress, it might develop as alopecia totalis (whole scalp hair loss) and even alopecia universalis (whole body hair loss). These clinical appearances really impacts patient's emotion and quality of life profoundly which results in the occurrence of various psychiatric disorders accordingly 14. Other than psychiatric disorders, a lot of reports have pointed out that AA is associated with cancers 15, 16, 17, 18, 19, 20, 21; however, no large‐scale study of different cancers in patients with AA has been conducted.

Alopecia areata is considered as a “benign” inflammatory process based on the histopathological findings of perifollicular T‐cell infiltrates without mucin deposition. The reactivation of CD8+ T cells to against hair follicle melanocytes and the following recruitment of CD4+ T‐lymphocytes to attack hair follicles via interferon‐*γ* producing resulting from the induction of major histocompatibility complex classes I and II expression on hair follicle epithelium were thought to be the major pathogenesis of AA. The accumulation of abnormal proliferation of T cells in AA and patients with mycosis fungoides (MF) and Sezary syndrome can manifest AA lesions without the presence of follicular mucinosis (FM) raised scientists’ interests in the risk of lymphoma in patient with AA 22. In the association between AA and MF, some researchers proposed that AA can be induced by the atypical or clonal T cells 17. Mediated by the Fas/Fas ligand pathway, these activated T cells in AA and MF might escape activation‐induced cell death and accumulate in the skin to form “abnormal” or “malignant” clonal or oligoclonal proliferations of CD4+ T cells ultimately 16, 23. The association with HLA‐DR5 and DQB1*03 alleles in both mycosis fungoides and AA further pointed out the overlapping between AA and FM/MF 23, 24. A significantly elevated risk for lymphoma observed in this study (SIR: 1.476), especially in female patients (SIR: 1.734) further supports those previous findings. Other than MF, AA has been referred to as a paraneoplastic syndrome of Hodgkin's lymphoma (HL) since early 1900s 17, 18, 25, 26, although remains limited to a few case studies. Even if the etiology of AA as a paraneoplastic syndrome of HL has not been fully elucidated, cellular immune responses impairment or the anergy occurred in HL were thought to be the possible causes, based on the findings that the number of circulating T lymphocytes (in particular, CD8+ suppressor T‐lymphocytes) are reduced in AA 13, 15, 17.

Upregulation of NKG2D ligands (NKG2DLs) along with the infiltration of cytotoxic subset of CD8+NKG2D+ T cells in AA patient is the major pathogenesis of AA 3, 4, 12. NKG2D and its ligand are known for their protective role in tumor immune surveillance. Combined with the immune defense against tumors effect achieved by NK cells 27, it would be easy to conclude that cancer risk should be lower in patient with AA. Our data seem support this speculation that the total cancer risk in AA patients is slightly lower than normal population, especially among male subjects. Refer to individual cancer, we further identified that the risk of NMSC, upper GI cancer, liver cancer, uterine, and cervix cancer are dramatically lower in patient with AA. However, the scenario is not that straightforward and easy. It has been noticed that there are different subsets of NK cells and some of them appeared lost or decreased cytotoxic or anti‐tumor activities, especially in breast and bladder cancer 28, 29, 30 which is compatible with our findings that AA patients have a dramatically increased incidence of breast and bladder cancer particularly in young individuals. In addition, more and more researches pointed out that NKG2D and its ligands are also exploited as tumor survival assets, enabling immune evasion and suppression, and quite possibly stimulation of tumor growth and malignant progression 31, 32, 33, 34, 35, 36. We think the paradoxical results of the cancer risk between AA and cancers might be due to the conflict role of the NKG2D receptor and its ligands among different cancers 35.

Giving that follicular melanocytes are the possible targets in AA according to the previous observation that nonpigmented hairs regrow first in areas of alopecia 37, 38, the occurrence of melanoma was expected to be lower in patients with AA. However, we did not see this trend in our result, which is compatible with previous study that the incidence of melanoma in AA patients is not significantly decreased 8. We think low incidence of melanoma in Taiwan is the possible reason to explain this result. One recent report pointed out the incidence of NMSC was decreased in patients with AA 8. Our study also showed the similar findings that NMSC is decreased in total population. Different to previous work which did not analyze the gender distinction, our data indicated that only male but not female AA patients had lower risk in NMSC formation. The incidence of skin cancer within female in Taiwan was much lower than male might explain this discrepancy because the case number is too low to reach the significance 39.

Autoimmune disorders were thought to be related with cancer formation. Exclusion of underlying autoimmune disorders did not affect the SIR among different cancers illustrated that the cancer risk in AA patients was not associated with their underlying autoimmune disorders.

Activation of JAK/STAT signaling pathway is recently identified as one of the most important pathogenesis of AA 12. Coincidentally, aberrant activation of JAK/STAT signaling confers malignant properties on cancer cells, which produces the strategy for drug development. JAK inhibitors which have been found to be effective in AA treatment recently 12 are utilized in couple clinical trials for cancer therapy 40. Based on this scenario, JAK inhibitor seems to kill two birds with one stone that it can control or prevent the cancer progression while AA was treated by it, especially for breast cancer 40.

There are a couple of limitations in this study. First, we cannot tell if the severity of AA will affect the cancer risk. Second, genetic and environmental factors are important in the pathogenesis of AA as well as in cancer formation, but the personal or family histories of patients were not available from the database.

In conclusion, this study carries out the first nationwide scale study of cancer risk in patients with AA. The association between cancer and AA is organ specific, and it is not related to the underlying autoimmune disorders in patients with AA. Although the overall cancer risk is not elevated in AA patients, AA patients might have increased risks in lymphoma, breast cancer, kidney, and bladder cancers, especially in female patients. We suggested a routine urine test for all AA patients for an early screening of tumors from kidney or urinary tract. In addition, scheduled physical and laboratory examination such as breast mammography and routine complete blood test for female patients.

## Conflict of Interest

The authors declare no financial or personal conflicts of interest.

## Supporting information


**Table S1.** The standardized incidence ratio of specific cancer risk in patients with alopecia area, stratified by gender and exclusion of autoimmune disorders.
