# Supplementary material for: Cancer risk in patients with alopecia areata: a nationwide population‐based matched cohort study
Source: Cancer Med. 2018 Mar 25;7(5):2153–9. doi: 10.1002/cam4.1448 (PMC5943418; doi:10.1002/cam4.1448)
Supplement: Supplementary file 1 — Table S1. The standardized incidence ratio of specific cancer risk in patients with alopecia area, stratified by gender and exclusion of autoimmune disorders. [file CAM4-7-2153-s001.docx]

**Supplementary table 1. The standardized incidence ratio of specific cancer risk in patients with alopecia area, stratified by gender and exclusion of autoimmune disorders**

|  | **All** | | |  | **Female** | | |  | **Male** | | |
| --- | --- | --- | --- | --- | --- | --- | --- | --- | --- | --- | --- |
|  | **N** | **SIR** | **95% C.I** |  | **N** | **SIR** | **95% C.I** |  | **N** | **SIR** | **95% C.I** |
| **Non hematologic cance**r | 1971 | 1.10 | 1.05-1.15 |  | 1074 | 1.26 | 1.19-1.34 |  | 897 | 0.95 | 0.89-1.01 |
| **Oral cavity** | 78 | 1.02 | 0.79-1.25 |  | 10 | 0.86 | 0.33-1.39 |  | 68 | 1.05 | 0.80-1.30 |
| **Nasopharynx and Pharynx** | 80 | 0.97 | 0.76-1.18 |  | 21 | 1.08 | 0.62-1.54 |  | 59 | 0.94 | 0.70-1.18 |
| **Upper GI** | 136 | 0.71 | 0.59-0.83 |  | 50 | 0.77 | 0.56-0.99 |  | 86 | 0.68 | 0.53-0.82 |
| **Colon** | 266 | 0.98 | 0.86-1.10 |  | 127 | 0.98 | 0.81-1.15 |  | 139 | 0.98 | 0.82-1.15 |
| **Liver** | 189 | 0.82 | 0.70-0.93 |  | 53 | 0.75 | 0.55-0.96 |  | 136 | 0.85 | 0.70-0.99 |
| **Lung** | 204 | 1.14 | 0.99-1.30 |  | 100 | 1.15 | 0.93-1.38 |  | 104 | 1.13 | 0.92-1.35 |
| **Thymoma** | 12 | 1.30 | 0.56-2.04 |  | 7 | 1.73 | 0.45-3.00 |  | 5 | 0.97 | 0.12-1.82 |
| **Connective tissue cancer and bone** | 21 | 1.00 | 0.57-1.42 |  | 11 | 1.13 | 0.46-1.80 |  | 10 | 0.88 | 0.33-1.43 |
| **Skin, melanoma** | 8 | 1.56 | 0.48-2.64 |  | 6 | 2.25 | 0.45-4.05 |  | 2 | 0.81 | 0.00-1.94 |
| **Skin, NMSC** | 30 | 0.60 | 0.38-0.81 |  | 19 | 0.78 | 0.43-1.12 |  | 11 | 0.43 | 0.17-0.68 |
| **Female breast** | 391 | 2.95 | 2.66-3.25 |  | 391 | 2.95 | 2.66-3.25 |  | 0 | - | - |
| **Male breast** | 1 | 1.07 | 0.00-3.16 |  | 0 | - | - |  | 1 | 1.07 | 0.00-3.16 |
| **Uterine and cervix** | 147 | 0.84 | 0.70-0.97 |  | 147 | 0.84 | 0.70-0.97 |  | 0 | - | - |
| **Prostate** | 59 | 1.27 | 0.95-1.59 |  | 0 | - | - |  | 59 | 1.27 | 0.95-1.59 |
| **Kidney and urinary bladder** | 109 | 2.88 | 2.34-3.42 |  | 38 | 3.07 | 2.09-4.05 |  | 71 | 2.78 | 2.14-3.43 |
| **Thyroid** | 101 | 1.12 | 0.90-1.33 |  | 76 | 1.06 | 0.83-1.30 |  | 25 | 1.31 | 0.80-1.82 |
| **Hematologic cancer** | 110 | 1.18 | 0.96-1.40 |  | 63 | 1.43 | 1.08-1.79 |  | 47 | 0.95 | 0.68-1.22 |
| **Lymphoma** | 73 | 1.53 | 1.18-1.88 |  | 44 | 1.91 | 1.34-2.47 |  | 29 | 1.17 | 0.75-1.60 |
| **Leukemia** | 37 | 0.81 | 0.55-1.07 |  | 19 | 0.91 | 0.50-1.32 |  | 18 | 0.72 | 0.39-1.06 |
